# True brachial artery aneurysm after arteriovenous fistula closure following renal transplantation: a case report and literature review

**DOI:** 10.1186/s40792-019-0724-4

**Published:** 2019-12-04

**Authors:** Satoshi Toyota, Kentaro Inoue, Shun Kurose, Shinichiro Yoshino, Ken Nakayama, Sho Yamashita, Koichi Morisaki, Tadashi Furuyama, Masaki Mori

**Affiliations:** Department of Surgery and Science, Graduate School of Medical Sciences, Kyushu University, 3-1-1, Maidashi, Higashi-ku, Fukuoka 812-8582 Japan

**Keywords:** Brachial artery aneurysm, Renal transplantation, Arteriovenous fistula

## Abstract

**Background:**

A brachial artery aneurysm (BAA) is a rare condition accounting for 5% of all peripheral arterial aneurysms. More cases of true BAAs after arteriovenous fistula (AVF) closure have been reported in the past two decades.

**Case presentation:**

A 60-year-old man who underwent AVF closure after renal transplantation had a true BAA on his left elbow that had grown within the past 6 months. We successfully performed an open repair with end-to-end anastomosis. No complications occurred for 1 year.

**Conclusions:**

High flow due to AVF and some collateral factors such as the use of steroids and immunosuppressants after renal transplantation, arteriosclerosis, and chronic mechanical stimulation might contribute to BAA formation.

## Background

A brachial artery aneurysm (BAA) is a rare condition accounting for 5% of all peripheral arterial aneurysms [1]. Most BAAs are pseudoaneurysms caused by trauma or iatrogenic complications [2, 3]; true BAAs are quite rare. The main etiologies of true BAAs are blunt trauma, atherosclerosis, infection, and vasculitis, and more than 50% of all patients with true BAAs have a history of blunt trauma [2]. A recently reported rare cause of true BAAs is arteriovenous fistula (AVF) closure after hemodialysis or renal transplantation [4, 5]. High flow due to AVF and essential drugs after transplantation, steroids, and immunosuppressants can also cause BAAs [4, 5]. The standard treatment for BAAs remains controversial because of their rarity and thus lack of detailed information.

This report describes a case of a true BAA after AVF closure following renal transplantation. The BAA was treated by excision and end-to-end brachial artery reconstruction. We also reviewed cases of idiopathic true BAAs and evaluated the etiology and optimal treatment for true BAAs.

## Case presentation

**Fig. 1:**
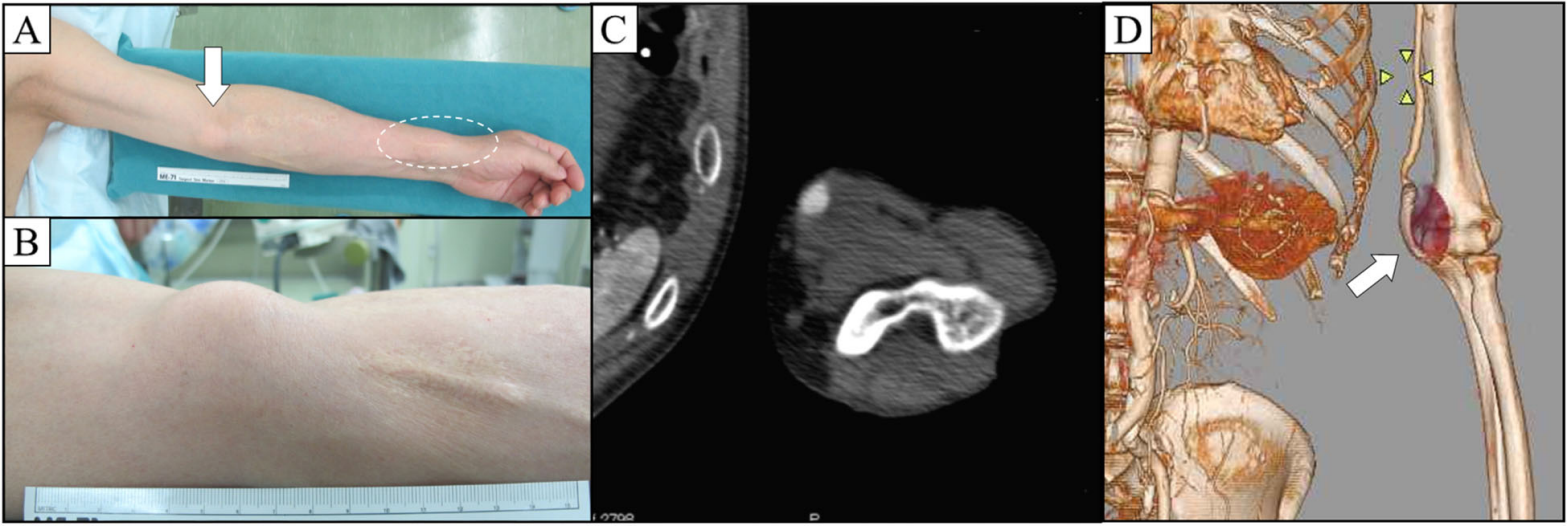
Preoperative imaging. **a**, **b** The left brachial mass was apart from AVF closure site (white dot circle). **c**, **d** Enhanced computed tomography showed a fusiform BAA with intramural thrombus and eccentrically patent lumen

A 60-year-old Japanese man presented with a left brachial mass that had developed during the past 6 months. The mass was 3.5 cm in size, pulsatile, and unaccompanied by pain, tenderness, or skin symptoms. The patient had started hemodialysis 27 years previously from a radiocephalic AVF on the left arm. He underwent cadaveric renal transplantation 15 years previously and had been administered immunosuppressive and steroid therapy (tacrolimus at 4 mg/day and prednisolone at 5 mg/day) to prevent renal rejection. The AVF was closed 4 years after renal transplantation. A BAA was diagnosed by enhanced computed tomography (CT), which showed a 35-mm-diameter fusiform BAA (Fig. 1c, d). Although an intraluminal thrombus was observed at the BAA, the distal blood flow was preserved.

**Fig. 2:**
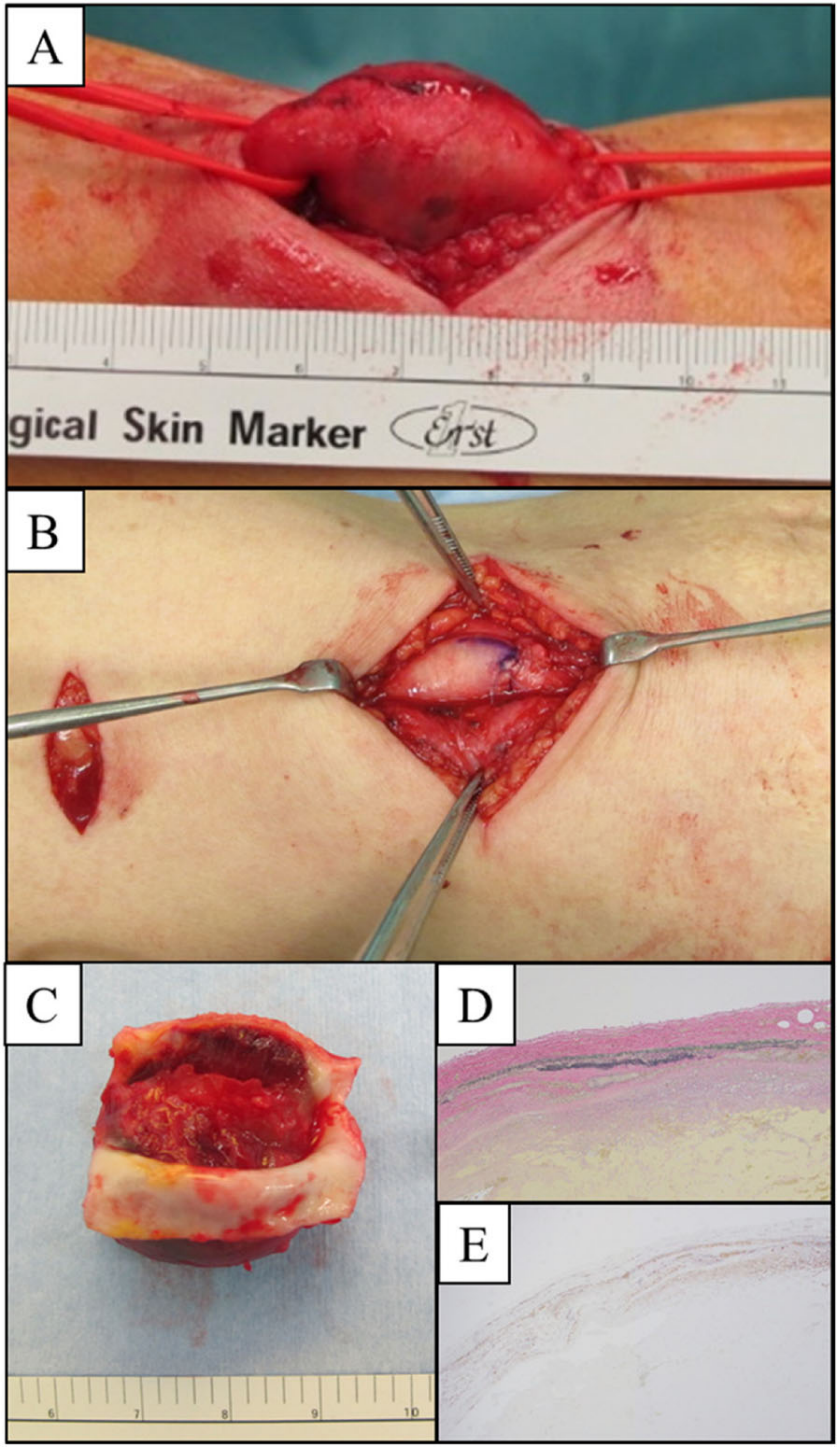
Operative findings. **a** The BAA was completely encircled. **b** End-to-end anastomosis was performed. **c** Solid thrombus was found in the BAA. Pathological findings. EV stain (**d**) and SMA (**e**) stain showed the well-maintained three-layer structure of arterial wall

The patient underwent aneurysm resection and open surgical revascularization because the aneurysm had gradually increased in size and limited the joint mobility. Under general anesthesia, the patient underwent excision of the BAA and end-to-end brachial artery reconstruction with 6–0 polypropylene sutures (Fig. 2a, b). The operative period was 1 h 21 min, and blood loss was minimal. The postoperative course was uneventful, and the patient was discharged 8 days postoperatively. The aneurysm was characterized by thickened vessel walls, and thrombosis was found in the lumen (Fig. 2c). Pathological examination showed a thickened tunica externa and thinned tunica intima and media. Internal elastic lamina was thinning and partially vanished but a three-layer structure was well-maintained (Fig. 2d, e); therefore, the BAA was diagnosed as a true aneurysm. It did not reveal typical arteriosclerotic. The patient was in good condition without recurrent symptoms 1 year postoperatively.

## Discussion

We have herein reported a true BAA following AVF closure after renal transplantation. The patient was treated by excision of the BAA and end-to-end brachial artery reconstruction.

Most true BAAs are caused by blunt trauma, atherosclerosis, infection, and vasculitis [2]. Regarding our patient, atherosclerosis might affect the formation of aneurysm, but it is not certain (pathological examination did not reveal typical arteriosclerotic, but CT findings showed the severe arteriosclerotic change in the abdominal aorta) and other histories of trauma or iatrogenic puncture ware absence. This idiopathic case without typical medical history is quite rare.

We searched PubMed for cases of true BAA using the keyword “true brachial artery aneurysm.” This search produced detailed reports of idiopathic true BAAs in 48 adults (Table 1). The patients’ mean age was 53.7 years (*n* = 46). Approximately 85% of the patients were men (39/46). The mean maximum diameter of the BAAs was 4.8 cm (*n* = 46). Interestingly, 41 patients (85%) had a medical history of AVF creation. Eugster et al. [6] said AVF side brachial artery generally expand after AVF creation and increases the blood flow of the brachial artery. The high flow increased shear forces which induced transverse tears in the elastic fibers of internal elastic membrane [7, 8]. And more, at the molecular level, active metabolism due to high flow and stress causes endothelial cells to produce superoxide ions and nitric oxide; these in turn form peroxynitrates that stimulate metalloproteinase, which breaks down the extracellular matrix and injures the internal elastic lamina [4, 5, 9, 10]. In fact, 3-D CT showed expanded left brachial artery which indicates high flow and pathological examination revealed internal elastic lamina was thinning and partially vanished.

**Table 1:** Literature review of cases of idiopathic true brachial artery aneurysm

Author*	Year	Number of patients	Age	Sex	AVF	Renal transplantion	Size (mm)	Symptom	Location	Treatment
Our case	2018	1	60	Male	Yes	Yes	35	No symptom	Apart from AVF	End-to-end anastomosis
Hale et al.	1994	1	35	Male	Yes	No	70	Distal emboli	Apart from AVF	GSV graft
Gray RJ et al.	1998	1	unknown	unknown	No	No	53	Thrombosis	Unknown	GSV graft
Nuguyen et al.	2001	1	51	Male	Yes	Yes	50	Paresthesia, distal emboli	Apart from AVF	GSV graft
Schunn CD et al.	2002	1	52	Male	Yes	No	140	Pain	Apart from AVF	Forearm vein interposition
Eugster et al.	2003	1	unknown	unknown	Yes	Unknown	unknown	Distal emboli	Apart from AVF	GSV graft
Battaglia et al.	2006	1	58	Male	Yes	Yes	50	Mild pain	Apart from AVF	PTFE
Ventura et al.	2006	1	63	Male	Yes	Yes	50	Pain	Apart from AVF	PTFE
Sultana et al.	2006	1	63	Male	Yes	Yes	37	Distal emboli	Apart from AVF	GSV graft
Chemla et al.	2010	5	51	Male	Yes	Yes	40	Pain	Apart from AVF	End-to-end anastomosis
			42	Male	Yes	No	40	No symptom	Apart from AVF	GSV graft
			73	Male	Yes	No	50	Pain	Apart from AVF	End-to-end anastomosis
			50	Female	Yes	Yes	80	Pain	Apart from AVF	GSV graft
			47	Male	Yes	Yes	50	No symptom	Apart from AVF	End-to-end anastomosis
Murphy et al.	2010	1	61	Male	Yes	Yes	30	Pain	Apart from AVF	PTFE
Omer et al.	2010	1	50	Female	No	No	40	Pulsatile mass	Unknown	GSV graft
Hudorović et al.	2010	1	77	Male	No	No	50	Painless swelling mass	Unknown	GSV graft
Alagaratnam et al.	2011	1	64	Female	No	No	34	Nerve compression paresthesia, swelling	Unknown	GSV graft
Dinoto et al.	2012	1	64	Male	Yes	Yes	84	Pain, paresthesia	Apart from AVF	PTFE
Shawon et al.	2012	1	33	Female	Yes	No	25	Pain, paresthesia	Apart from AVF	GSV graft
Sydney et al.	2012	1	37	Male	Yes	No	44	No symptom	Apart from AVF	GSV graft
Bassir et al.	2012	1	67	Male	No	No	20	Pain	Unknown	Thrombectomy + GSV interposition
Ettore et al.	2012	1	61	Male	Yes	Yes	150	Pain, paresthesia, swelling mass	Apart from AVF	Aneurysmectomy and vein grafting
Khalid et al.	2014	3	44	Female	Yes	Yes	29	Pain	Apart from AVF	GSV graft
			50	Male	Yes	Yes	21	Distal emboli	Apart from AVF	GSV graft
			75	Female	Yes	Yes	53	Pain	Apart from AVF	Ligation of feeding vessel
Sandeep et al.	2014	2	60	Male	Yes	Yes	30	Pain, aneurysm thrombus	Apart from AVF	GSV graft
			63	Male	Yes	Yes	30	Pain	Apart from AVF	GSV graft
De Santis et al.	2014	1	47	Male	Yes	Yes	unknown	Painful pulsatile mass	Near AVF	Direct arterial wall suture
Marconi et al.	2015	1	61	Male	Yes	Yes	200	No symptom	Apart from AVF	ePTFE grafting
Emily CC et al.	2105	1	48	Male	Yes	Yes	20	Paresthesia, distal emboli	Apart from AVF	Cephalic vein graft
Nishimura et al.	2016	1	65	Male	Yes	No	40	Ulceration of fingers, microembolization	Unknown	Aneurysmectomy and GSV grafting
Yuan et al.	2016	1	38	Male	No	No	35	Painful pulsatile mass	Apart from AVF	GSV interposition
Teixeira et al.	2017	10	mean 52 37–63	Male 9 Female 1	Yes 10	Yes 9 No 1	mean 37.5 17.5–64	Pain and pulsatile mass 6 cases ischemia microembolization 3 cases acute ischemia aneurysm thrombosis 1 case	Unknown	aneurysmectomy and vein grafting 9 caseaneurysmectomy and ePTFE grafting 1case
Anup et al.	2017	1	59	Male	Unknown	Unknown	48	Acute ischemia aneurysm thrombosis	Apart from AVF	Aneurysmectomy and GSV grafting
Fendri et al.	2017	5	47	Male	Yes	Yes	45	Pain	Apart from AVF	GSV graft
			37	Male	Yes	Yes	30	Pain	Apart from AVF	GSV graft
			40	Male	Yes	Yes	18	Pain	Apart from AVF	Femoral artery graft
			43	Male	Yes	Yes	27.3	No symptom	apart from AVF	No surgery
			76	Male	Yes	Yes	30	Pain	Apart from AVF	GSV graft

*References for this table were summarized in the Additional file 1

In addition to this, some collateral factors might contribute to the aneurysm formation. One was steroids and immunosuppressants’ use after renal transplantation. According to our review, 22 patients (46%) underwent AVF closure after renal transplantation. Generally, steroids are suggested to promote the formation and enlargement of arterial aneurysms [11]. They not only impair glucose tolerance and exacerbate arteriosclerosis, thus indirectly promoting aneurysm formation, but also directly cause tissue fragility that mainly affects small- to medium-sized arteries [12]. Immunosuppressants are also suggested to provide a synergistic effect on the damage caused by steroids [12]. In our case, the patient underwent renal transplantation and began treatment with a steroid (prednisolone at 5 mg/day) and immunosuppressant (tacrolimus at 4 mg/day) 15 years before the emergence of the BAA. Another factor was arteriosclerotic. The pathological examination did not reveal typical arteriosclerotic change, but CT findings showed the severe arteriosclerotic change in the abdominal aorta, so it was very likely that the brachial artery got some arteriosclerotic effect and damaged. And more, Marconi et al. [13] and Nishimura et al. [14] reported true BAA located near the elbow like our case. Chronic mechanical stimulation, an elbow joint movement might also promote damages to the brachial artery.

Although BAAs are rare, it is often difficult to determine whether a BAA itself is symptomatic because 33% of BAAs are associated with complications such as pain, edema, neuropathy, distal limb ischemia, and aneurysm rupture [2]. In the present review, only 7 patients (15%) were asymptomatic or showed only a swelling mass. Importantly, 12 of 13 relatively small aneurysms (< 35 mm) were symptomatic, and the size of the aneurysm did not seem to be correlated with the patient’s symptoms. Pain was the most frequent symptom (56%, 27/48), and thrombi/emboli and paresthesia were found in 29% (14/48) and 8% (4/48) of patients, respectively. Although our review did not include cases of rupture, rupture of a BAA can be an indication for upper limb amputation [15]. Fundamentally, therefore, BAAs should be treated independent of size, and careful follow-up is required when an observation is selected.

With respect to treatment, 47 patients (97%) in our review underwent surgical treatment (Table 1). Surgical treatment is often selected because the stenting of joints, including the elbow, is not favorable. Interposition with a native vessel graft was widely applied (75%, 36/48), and the great saphenous vein was used in most cases. In case of not available of saphenous vein, femoral vein or polytetrafluoroethylene was used. However, the use of conduit has some risk such as graft occlusion or size mismatch between the host artery and graft [4]. End-to-end anastomosis can be a more physiological and safe brachial artery reconstruction technique. Further studies are required to reveal the detailed pathology and optimal management of BAAs following AVF closure after renal transplantation.

## Conclusion

High flow due to AVF and some collateral factors such as the use of steroids and immunosuppressants after renal transplantation, arteriosclerosis, and chronic mechanical stimulation might contribute to true BAA formation. Careful follow-up is desirable for such a case.

## Supplementary information

**Additional file 1.** References for the literature review.

## Data Availability

None (because our manuscript is a case report).

## Abbreviations

AVF: Arteriovenous fistula
BAA: Brachial artery aneurysm
